# Supplementary material for: Automated incorporation of pairwise dependency in transcription factor binding site prediction using dinucleotide weight tensors
Source: PLoS Comput Biol. 2017 Jul 28;13(7):e1005176. doi: 10.1371/journal.pcbi.1005176 (PMC5550003; doi:10.1371/journal.pcbi.1005176)
Supplement: S1 Text — (PDF) [file pcbi.1005176.s001.pdf]

Supporting Text

Automated Incorporation of Pairwise Dependency in Transcription Factor Binding Site Prediction Using Dinucleotide Weight Tensors

Saeed Omidi, Mihaela Zavolan, Mikhail Pachkov, Jeremie Breda, Severin Berger
Erik van Nimwegen
Biozentrum, the University of Basel, and Swiss Institute of Bioinformatics
Klingelbergstrasse 50/70, 4056-CH, Basel, Switzerland,
email: erik.vannimwegen@unibas.ch

Contents

1 Calculating posterior probabilities for the pairwise dependencies 1

2 Rescaling of the dependency matrix 3

3 Scoring of partial site matches 4

4 DWTs with only adjacent dependencies: The ADJ model 5

5 Training and testing the PIM model 5

6 Training and test the FMM model 5

7 Table 1 6

1 Calculating posterior probabilities for the pairwise dependencies

As part of the dilogo we calculate, for each pair of positions (i, j) the posterior probability $P(i, j|S)$, that a direct dependency between exists between positions i and j, given the sequence alignment S. As we have shown previously [1], the posterior probability $P(i, j|S)$ is given by of the sum of $P(S|\pi)$ over all spanning trees in which the edge (i, j) occurs, divided by $P(S)$, i.e. $P(S|\pi)$ summed over all trees, irrespective of the occurrence of the edge (i, j). That is, we have

$$P(i, j|S) = \frac{\sum_{\pi|(i,j)\in\pi} P(S|\pi)}{\sum_{\pi} P(S|\pi)}, \tag{1}$$

and Fig. 1 illustrates all the topologies that contribute to the sum in the numerator and denominator of this ratio for a sequence of length 4.

As we also derived previously [1], this posterior can be calculated by defining a new $(l-1)$ by $(l-1)$ matrix $R^{(i,j)}$ in which the two nodes i and j have been 'contracted' into a single node (i, j). The entries for the matrix elements involving this node are given by

$$R^{(i,j)}_{(i,j)k} = R_{ik} + R_{jk}, \tag{2}$$

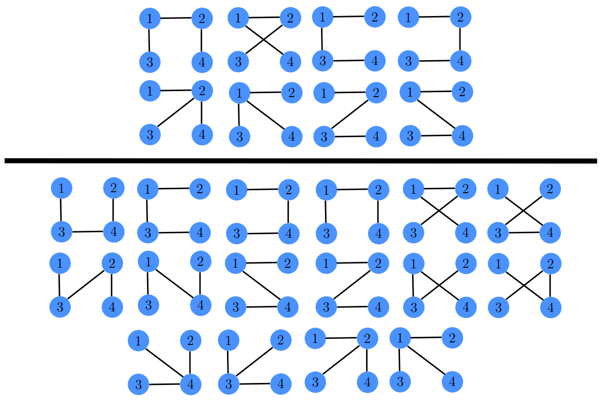

Supplementary Figure 1: Illustration of the calculation of the posterior probability that positions 1 and 2 are directly connected, for the simple case of sequences of length 4. Each position is represented by a node in the possible spanning tree graphs π. In the numerator are all trees in which the edge $(1, 2)$ appears, and in the denominator are all possible spanning trees.

whereas

$$R^{(i,j)}_{kl} = R_{kl}, \tag{3}$$

for all other nodes. Using this contracted matrix $R^{(i,j)}$, the posterior is given by

$$P(i, j|S) = \frac{R_{ij} D(R^{(i,j)})}{D(R)}. \tag{4}$$

Note, however, that these calculations assume that each position has 1 parent that it depends on, i.e. it is impossible for a position not to have a dependency. While this is a reasonable approximation for applications where dependencies are common, in our case there are a considerable number of motifs where the PSWM appears to be an excellent approximation, i.e. there is almost no evidence for dependencies, and forcing each position to have a dependency is inappropriate. To account for this, we extended the calculations to not just sum over all possible spanning trees, but over all possible *forests* of the positions in a site. Spanning forests consist of all factorizations of the positions into one or more trees. Equivalently, each position in the site can either have zero or 1 other position that it depends on. We assign a prior to the space of forests in proportion to the number of edges occurring in the forest, i.e. if ϕ is a forest of the l positions with n edges in total, we assign a prior probability $P(\phi) \propto \rho^n (1 - \rho)^{l-1-n}$, where ρ can be interpreted as the prior probability that a given position has a dependency. All calculations the apply to sums over spanning trees can be easily extended to sums over forests by replacing the matrix R with a matrix Q given by

$$Q_{ij}(\rho) = R_{ij}\rho + (1 - \rho). \tag{5}$$

The contracting of the edge works exactly the same for matrix $Q(\rho)$ as for matrix R and equation (4) is replaced by

$$P(i, j|S) = \frac{\rho R_{ij} D(Q^{(i,j)}(\rho))}{D(Q(\rho))}. \tag{6}$$

Note that the expression $D(Q(\rho))$ corresponds to the the log-likelihood of the sequences S given

ρ. Thus, to calculate the posterior probabilities of dependency for a given DWT, we first determine the value ρ_* that maximizes $D(Q(\rho))$ and then calculate posteriors using equation (6) with ρ set to ρ_*.

2 Rescaling of the dependency matrix

When the pair-counts $n^{ij}_{\alpha\beta}$ are large, the entries R_{ij} of the dependency matrix R may range over many orders of magnitude. When this happens, the calculation of the determinant $D(R)$ may become numerically unstable. As far as we are aware, there is no principled method for avoiding this numerically instability of determinant calculations and we therefore rely on an *ad hoc* procedure for ensuring the determinant calculation is numerically stable. In particular, when the largest and smallest values of the R matrix, call them $R_{\max}$ and $R_{\min}$, vary by more than a factor e^k we rescale all entries in log-space the transformation

$$\log[R_{ij}] \rightarrow \log[\tilde{R}_{ij}] = \alpha \log[R_{ij}], \tag{7}$$

where $\alpha = k/(\log[R_{\max}] - \log[R_{\min}])$. Note that, consequently, the entries of the transformed matrix span a range of e^k. In this study we chose $k = 25$, i.e. the maximum ratio between the largest and smallest entry of the rescaled $\tilde{R}$ is $e^{25} \approx 7 * 10^{10}$. Note that matrix entries for which the dependent and independent models have equal likelihood, i.e. when $R_{ij} = 1$, are invariant under this rescaling transformation.

As explained in the main text, calculation of the conditional probability $P(s|S)$ involves the ratio of determinants $D(R(s,S))/D(R(S))$, i.e. see equation (9) of the main text. When both the matrices $R(S)$ and $R(s,S)$ are naively rescaled to $\tilde{R}(s,S)$ and $\tilde{R}(S)$ according to the formula (7), then the resulting $P(s|S)$ may no longer be precisely normalized, i.e. the sum $\sum_s P(s|S)$ over all possible sequence segments s is no longer strictly 1. However, for the stability of the iterative motif finding procedure it is essential that the conditional probabilities $P(s|S)$ are strictly normalized. To ensure this we adapted the rescaling procedure as follows.

Note that the conditional probability $P(s|S)$ can also be written as

$$P(s|S) = \sum_{\pi} P(\pi|S) P(s|S,\pi), \tag{8}$$

with

$$P(s|S,\pi) = P(s_r|S) \prod_{i \neq r} P(s_i|s_{\pi(i)}, S), \tag{9}$$

the conditional probabilities $P(s_i|s_j,S)$ are given by

$$P(s_i|s_j,S) = \frac{P(s_i,s_j|S)}{P(s_j|S)} = \frac{n^{ij}_{s_i s_j} + \lambda'}{n + 16\lambda'} \left[\frac{n^j_{s_j} + \lambda}{n + 4\lambda}\right]^{-1}, \tag{10}$$

and the posterior probability $P(\pi|S)$ of the spanning tree π given alignment S is given by

$$P(\pi|S) = \frac{\prod_{(i,j)\in\pi} R_{ij}(S)}{\sum_{\pi'} \prod_{(i,j)\in\pi'} R_{ij}(S)} = \frac{\prod_{(i,j)\in\pi} R_{ij}(S)}{D(R(S))}. \tag{11}$$

That is, the probability $P(s|S)$ can be written as a weighted sum over all possible spanning trees π of the conditional probability $P(s|S,\pi)$ given the sequences in S and the spanning tree π, weighing each spanning tree with its posterior probability $P(\pi|S)$ given the sequences in S. To ensure numerical stability while retaining the strict normalization of $P(s|S)$ we only rescale the entries of R in the expression $P(\pi|S)$. That is we replace $P(\pi|S)$ with

$$\tilde{P}(\pi|S) = \frac{\prod_{(i,j)\in\pi} \tilde{R}_{ij}(S)}{D(\tilde{R}(S))}, \tag{12}$$

and substitute this in equation (8). This corresponds to calculating the conditional probabilities $P(s|S,\pi)$ exactly for each spanning tree π, while letting the rescaling only affect the relative probabilities $P(\pi|S)$ of the different spanning trees in the sum.

Finally, note that if we define the new matrix

$$\tilde{R}(s,S) = \tilde{R}_{ij}(S)\frac{(n^{ij}_{s_i s_j}+\lambda')(n+4\lambda)}{(n^i_{s_i}+\lambda)(n^j_{s_j}+\lambda)}, \tag{13}$$

then equation (8) can be rewritten as

$$P(s|S) = \frac{D(\tilde{R}(s,s))}{D(R(S))}\prod_{i=1}^{l}\frac{n^i_{s_i}+\lambda}{n+4\lambda}, \tag{14}$$

i.e. just as equation (9) in the main text.

3 Scoring of partial site matches

Here we derive an approximation for scoring sequence segments that contain one or more N (i.e. unknown) nucleotides. Formally, let x be a sequence segment that contains one or more N nucleotides and let $e^{E(x)} = P(x|M)/P(x|B)$ correspond to the score of this degenerate sequence. Formally, $P(x|M)/P(x|B)$ corresponds to the average of $P(s|M)/P(s|B)$ over all sequence segments s that are consistent with x, and weighing each possible segment s with probability proportional to its probability under the background model, i.e.

$$\frac{P(x|M)}{P(x|B)} = \sum_{s\in x} P(s|x)\frac{P(s|M)}{P(s|B)}, \tag{15}$$

where by a small abuse of notation we also use x to represent the set of sequence segments consistent with x and $P(s|x)$ is given by

$$P(s|x) = \frac{P(s|B)}{\sum_{s'\in x} P(s'|B)}. \tag{16}$$

Combining these equations we find

$$\frac{P(x|M)}{P(x|B)} = \frac{\sum_{s\in x} P(s|M)}{\sum_{s\in x} P(s|B)}. \tag{17}$$

For the PSWM model the scores are given by simple products, i.e. $P(s|M) = \prod_{i=1}^{l} w^i_{s_i}$ and $P(s|B) = \prod_{i=1}^{l} b_{s_i}$. For each position i in sequence x that is N, the sum over all s involves a sum over all possible values that s_i can take. Since $\sum_\alpha w^i_\alpha = 1$, we have

$$\sum_{s\in x}\prod_{i=1}^{l} w^i_{s_i} = \prod_{i|s_i\neq N} w^i_{s_i}\prod_{i|s_i=N}\left[\sum_\alpha w^i_{s_i}\right] = \prod_{i|s_i\neq N} w^i_{s_i}, \tag{18}$$

i.e. the contribution of all positions i where $s_i = N$ just disappears from the sum. The same applies to the probability $P(x|B)$ and, consequently, the score $P(x|M)/P(x|B)$ is simple given by the product of contributions from all letters that are not N:

$$\frac{P(x|M)}{P(x|B)} = \prod_{i|s_i\neq N}\frac{w^i_{s_i}}{b_{s_i}}. \tag{19}$$

As we saw in equation (8) above, under the DWT model the probability $P(s|S)$ can be written as a weighted sum over spanning trees π, of the conditional probabilities $P(s|S,\pi)$ given a spanning π. In turn, the probabilities $P(s|S,\pi)$ can be written as a product over conditional probabilities $P(s_i|s_j,S)$ for each base s_i given its parent base s_j, i.e equation (9), and the conditional probability can be written as the product of the PSWM condition, and a factor that incorporates the effect of the dependency

$$P(s_i|s_j,S) = P(s_i|S)\frac{P(s_i,s_j|S)}{P(s_i|S)P(s_j|S)} = \left[\frac{n^i_{s_i}+\lambda}{n+4\lambda}\right]\left[\frac{(n^{ij}_{s_is_j}+\lambda')(n+4\lambda)}{(n^i_{s_i}+\lambda)(n^j_{s_j}+\lambda)}\right]. \qquad (20)$$

Note that the second factor on the right is precisely the factor by which the matrix $\tilde{R}(S)$ is multiplied to obtain $\tilde{R}(s,S)$ in equation (13). Finally, we saw that for the PSWM case, the score for sequences containing N nucleotides are obtained simply by only including the contributions from all nucleotides that are not N in the product over positions. In other words, the contribution $P(s_i|S)$ is set to 1 for positions i where $s_i = N$. This generalizes in a straight-forward way to the DWT case. In particular, the whenever letter $s_i = N$, we set $P(s_i|s_j,S) = 1$, which is equivalent to setting both $P(s_i|S) = 1$, and the factor $P(s_i,s_j|S)/(P(s_i|S)P(s_j|S)) = 1$. That is, to obtain matrix $\tilde{R}(s,S)$ of equation (13), we only multiply $\tilde{R}_{ij}(S)$ by the factor $P(s_i,s_j|S)/(P(s_i|S)P(s_j|S))$ when neither s_i nor s_j are N.

4 DWTs with only adjacent dependencies: The ADJ model

To assess the contribution of distal dependencies to the motif finding we investigated the performance of a restricted DWT model in which only dependencies between neighboring positions are allowed, which we call the adjacent (ADJ) model. Instead of summing over all spanning trees π, in the adjacent model each position i is only allowed to depend on the immediately adjacent positions $(i-1)$ and $(i+1)$. Restricting the sum over spanning trees in this way can be easily accomplished by simply setting $R_{ij} = 0$ whenever $i \neq (j+1)$ and $i \neq (j-1)$. That is, only the entries with $i = j+1$ and $i = j-1$ are retained.

5 Training and testing the PIM model

To train a motif the PIM model of Santolini *et al.* [2] requires an initial PSWM motif and, for a motif of length l, all l-mers occurring in the training data. Besides using the exact same training and test data for the 121 ChIP-seq datasets, we made sure train the PIM model starting from the exact same PSWM models as were used as a starting points to train the DWT models. However, since the method calculates statistics over all l-mers, and this becomes intractable for long motifs, e.g. $l = 20$, we needed to prune long motifs. Thus, whenever the initial PSWM motif was longer than PIM's default length of $l = 12$, we pruned the PSWM to the 12 consecutive columns with the highest information content. In addition, while PIM's motif training typically finished within half an hour, some datasets took many hours, and for 3 of the 121 datasets the training had not converged after several weeks of running. Time constraints necessitated us to terminate these runs and we thus did not obtain PIM results for 3 of the 121 datasets. We set the average precision to 0.2, i.e. equal to random performance, for these 3 datasets.

We adapted the PIM MATLAB code to use the trained model to calculate binding energies $E(s)$ for each sequence segment s occurring in the test set and we calculated total binding energies $E(S) = \log[\sum_{s\in s} e^{E(s)}]$ for each training sequence S in the exact same manner as for the DWT models.

6 Training and test the FMM model

The FMM method of Sharon *et al.* [3] differs from the other methods in that it does not require an initial PSWM motif (or a motif length), but in contrast to the other algorithms it requires not only a set of

positive sequences but also a set of negative sequences. For this we used a set of 2000 random sequences with the same dinucleotide content as the input sequences, i.e. just as the decoy sequences for testing were created. Because all other methods were asked to only infer one motif, we also instructed the FMM algorithm to infer a single motif.

Eilon Sharon graciously provided us with a python script that calculates FMM scores for every sequence segment s in the input sequences and we used this to calculate, for each sequence S in the test set, a total binding energy $E(S)$ from the binding energy of each segment s.

For 2 datasets the FMM model did not report a motif, presumably because it failed to detect any statistically significant sequence patterns, and we set the average precision to 0.2 for these 2 datasets.

7 Table 1

Combinations of HT-SELEX and ENCODE ChIP-seq dataset that were analyzed. The IDs in the first column each correspond to a dataset from [4] and the descriptions in the second column correspond to ENCODE ChIP-seq datasets (see crunch.unibas.ch/ENCODE_REPORTS/ for links to the processed and raw input data).

HT-SELEX dataset	ChIP-seq dataset
IRF4_TCAAGG20NCG_AD	Myers_HudsonAlpha-BG_1_2-IRF4
MEF2A_TAATAG20NTA_Q	Myers_HudsonAlpha-BG_8-MEF2A
BHLHE41_TGTGCT20NCGG_AD	Snyder_Stanford-IggMus-BHLHE
EBF1_TATAAG20NCG_AC	Snyder_Stanford-StandardControl-EBF1
BATF3_TAAGAC20NAGA_AC	Myers_HudsonAlpha-BG_1_2-BATF
ETS1_TGTAAA20NGA_AF	Myers_HudsonAlpha-BG_8-ETS1
YY1_TCCGGC20NCG_AC	Myers_HudsonAlpha-BG_4_8-YY1
YY1_TCCGGC20NCG_AC	Snyder_Farnham_USC-StandardControl-YY1
BHLHE23_TATATC20NCG_Y	Snyder_Stanford-IggMus-BHLHE
ELK1_TCGGAA20NAGT_AG	HeLaS3_Snyder_Stanford-IggRab-ELK1
ELK1_TCGGAA20NAGT_AG	Snyder_Stanford-IggMus-ELK1
POU2F2_TGACAG20NGA_AC	Myers_HudsonAlpha-BG_1_5-POU2
POU2F2_TGACAG20NGA_AC	Myers_HudsonAlpha-BG_1-POU2
RFX3_TGGCTT20NGA_AC	Snyder_Stanford-IggMus-RFX
GABPA_TGGCCC20NCCT_AG	Myers_HudsonAlpha-BG_6_7-GABP
CEBPB_TCAACC20NCAA_W	Myers_HudsonAlpha-BG_10-CEBP
ZNF143_TGCAAG20NCG_V	Snyder_Stanford-StandardControl-ZNF143
ZNF143_TGCAAG20NCG_V	HeLaS3_Snyder_Stanford-IggRab-ZNF143
MAX_TGACCT20NGA_Y	Snyder_Stanford-IggMus-MAX
MAX_TGACCT20NGA_Y	HeLaS3_Snyder_Stanford-IggRab-MAX
NFKB2_TTCAAT20NGA_R	Snyder_Stanford-IggRabTNFa-NFKB
E2F4_AGCAG14N_U	Snyder_Stanford-IggMus-E2F4
POU2F1_TCTTTC20NGA_AC	Myers_HudsonAlpha-BG_1_5-POU2
POU2F1_TCTTTC20NGA_AC	Myers_HudsonAlpha-BG_1-POU2
CTCF_full_AJ_TAGCGA20NGCT	Snyder_Stanford-StandardControl-CTCF
CTCF_full_AJ_TAGCGA20NGCT	Bernstein_BroadInstitute-StandardControl-CTCF
CTCF_full_AJ_TAGCGA20NGCT	Crawford_Iyer_UTAustin-StandardControl-CTCF
CTCF_full_AJ_TAGCGA20NGCT	Stamatoyannopoulous_UW-StandardControl-CTCF
ELK1_TGAGTG20NTGA_AG	HeLaS3_Snyder_Stanford-IggRab-ELK1

ELK1_TGAGTG20NTGA_AG	Snyder_Stanford-IggMus-ELK1
NRF1_TAGCGA20NCG_AC	HeLaS3_Snyder_Stanford-IggMus-NRF1
NRF1_TAGCGA20NCG_AC	Snyder_Stanford-IggMus-NRF1
TCF3_TACCCG20NCCC_Y	Myers_HudsonAlpha-BG_4_5-TCF3
CEBPG_TAAAAT20NCG_AC	Myers_HudsonAlpha-BG_10-CEBP
RUNX3_TCTCCC20NGA_AE	Myers_HudsonAlpha-BG_10-RUNX3
SRF_TGGAAT20NAAT_W	Myers_HudsonAlpha-BG_8-SRF
SRF_TGGAAT20NAAT_W	Myers_HudsonAlpha-BG_6_7-SRF
MAFK_TTAAAG20NTA_AE	Snyder_Stanford-IggMus-MAFK
MAFK_TTAAAG20NTA_AE	HeLaS3_Snyder_Stanford-IggRab-MAFK
PRDM1_TTGAGG20NGAT_AE	HeLaS3_Snyder_Stanford-IggRab-PRDM1
NFE2_TGTAGG20NGA_AC	Snyder_Stanford-StandardControl-NFE2
NFATC1_TTCGTA20NTGC_AE	Myers_HudsonAlpha-BG_10-NFATC1
IRF3_TCCTAA40NATC_AI	HeLaS3_Snyder_Stanford-IggRab-IRF3
IRF3_TCCTAA40NATC_AI	Snyder_Stanford-IggMus-IRF3
USF1_TGACGA20NGCA_Z	Myers_HudsonAlpha-BG_6_7-USF1

References

[1] Burger L, van Nimwegen E. Disentangling Direct from Indirect Co-Evolution of Residues in Protein Alignments. PLoS Comput Biol. 2010;6(1):e1000633.

[2] Santolini M, Mora T, Hakim V. Beyond position weight matrices: nucleotide correlations in transcription factor binding sites and their description. arXiv:13024424v1. 2013;.

[3] Sharon E, Lubliner S, Segal E. A feature-based approach to modeling protein-DNA interactions. PLoS Comput Biol. 2008;4(8):e1000154.

[4] Jolma A, Yan J, Whitington T, Toivonen J, Nitta KR, Rastas P, et al. DNA-binding specificities of human transcription factors. Cell. 2013;152(1-2):327–39.
